# Lobectomy for primary lung cancer: a comparison of perioperative and postoperative outcomes between robot-assisted thoracic surgery and video-assisted thoracic surgery

**DOI:** 10.1007/s00595-025-03000-6

**Published:** 2025-02-17

**Authors:** Harushi Ueno, Yoshito Imamura, Shoji Okado, Yuji Nomata, Hiroki Watanabe, Yuta Kawasumi, Keita Nakanishi, Yuka Kadomatsu, Taketo Kato, Shota Nakamura, Tetsuya Mizuno, Toyofumi Fengshi Chen-Yoshikawa

**Affiliations:** Department of Thoracic Surgery, Nagoya University Graduate School of Medicine, 65 Tsurumai-Cho, Showa-Ku, Nagoya, 466-8550 Japan

**Keywords:** Lung cancer, Lobectomy, Robot-assisted thoracic surgery (RATS), Video-assisted thoracic surgery (VATS), Perioperative outcomes, Postoperative outcomes, Long-term survival

## Abstract

**Purpose:**

This study compared the peri- and postoperative outcomes of robot-assisted thoracic surgery (RATS) and video-assisted thoracic surgery (VATS) lobectomy for primary lung cancer.

**Methods:**

This retrospective cohort study included patients who underwent RATS or VATS at Nagoya University Hospital between April 2018 and March 2022. Propensity score matching was used to balance patient characteristics between the two groups. The following long-term outcomes were assessed: the 3-year overall survival (OS), causes of death, 3-year disease-free survival (DFS), and recurrence patterns. Various peri- and postoperative outcomes were examined as short-term outcomes.

**Results:**

After propensity score-matching, 137 patients were included in each group. RATS was associated with a longer operative time (median 180 vs. 144 min, p < 0.001), less blood loss (median 5 vs. 12 ml, p = 0.005), and a lower rate of conversion to open thoracotomy (1 [0.7%] vs. 10 [7.4%], p = 0.010) than VATS. The 3-year OS and DFS were comparable between the groups.

**Conclusion:**

In lobectomy for lung cancer, RATS demonstrated long-term outcomes that were comparable to those of VATS. Although RATS has a longer operative time, it is associated with less blood loss and a lower conversion rate to open thoracotomy than VATS, suggesting that it is a beneficial surgical approach for patients.

**Supplementary Information:**

The online version contains supplementary material available at 10.1007/s00595-025-03000-6.

## Introduction

Lobectomy is the standard surgical procedure for primary lung cancer treatment. Since the advent of video-assisted thoracic surgery (VATS) in the 1990s, it has gradually become the standard treatment for early-stage primary lung cancer. Compared with open thoracotomy, VATS offers several advantages, such as reduced postoperative complications, less pain, shorter hospital stays, faster recovery of the physical function, and oncologic outcomes that are comparable to or even superior to those of open surgery. However, VATS also has limitations, including restricted movement, fulcrum effect, challenges with fine movements, and amplification of hand tremors.

Recently, the concept of robot-assisted thoracic surgery (RATS) has emerged in the field of thoracic surgery. RATS provides surgeons with high-definition visualization and enhanced dexterity, thereby facilitating precise manipulation, delicate handling of blood vessels, and meticulous lymph node dissection. Consequently, the use of RATS is increasing globally. With a growing body of evidence, several meta-analyses comparing RATS with VATS have been published [1–7], suggesting that RATS achieves perioperative and postoperative outcomes comparable to those of VATS. Studies in Japan comparing RATS and VATS have also demonstrated comparable perioperative outcomes [8, 9]. However, since RATS lobectomy for primary lung cancer only recently gained insurance coverage in Japan in 2018, data on long-term outcomes and comprehensive evaluations encompassing such results remain elusive.

In this retrospective study, we compared the perioperative, postoperative, and long-term outcomes of RATS and VATS lobectomy for lung cancer at our institution, at which we initiated RATS in 2018.

## Methods

All procedures were performed in accordance with the ethical standards of the responsible committee on human experimentation (institutional and national) and the 1964 Declaration of Helsinki and its subsequent revisions. The study protocol was approved by the institutional review board of Nagoya University School of Medicine (2020-0375, October 14, 2020). The requirement for informed consent was waived owing to the retrospective nature of the study.

This retrospective comparative cohort study included patients who underwent RATS or VATS lobectomy for primary lung cancer at Nagoya University Hospital between April 2018 and March 2022. The surgical approach of either open thoracotomy or minimally invasive surgery (RATS and VATS) was determined at a multidisciplinary thoracic surgery conference, considering factors such as the tumor size and location, patient history, and comorbidities. Cases that resulted in R1/2 resection, those that were not resected due to pleural dissemination, and those that underwent simultaneous bilateral surgery were excluded from this study. In addition, 19 patients who underwent single-port VATS were excluded because this approach was selectively used for early-stage lung cancer without lymph node dissection and a well-segmented lung at our institution. During the study period, patients with cN0 tumors or those anticipated to require vascular reconstruction, bronchial reconstruction, or chest wall resection were deemed ineligible for minimally invasive surgery at our institution.

**Fig. 1:**
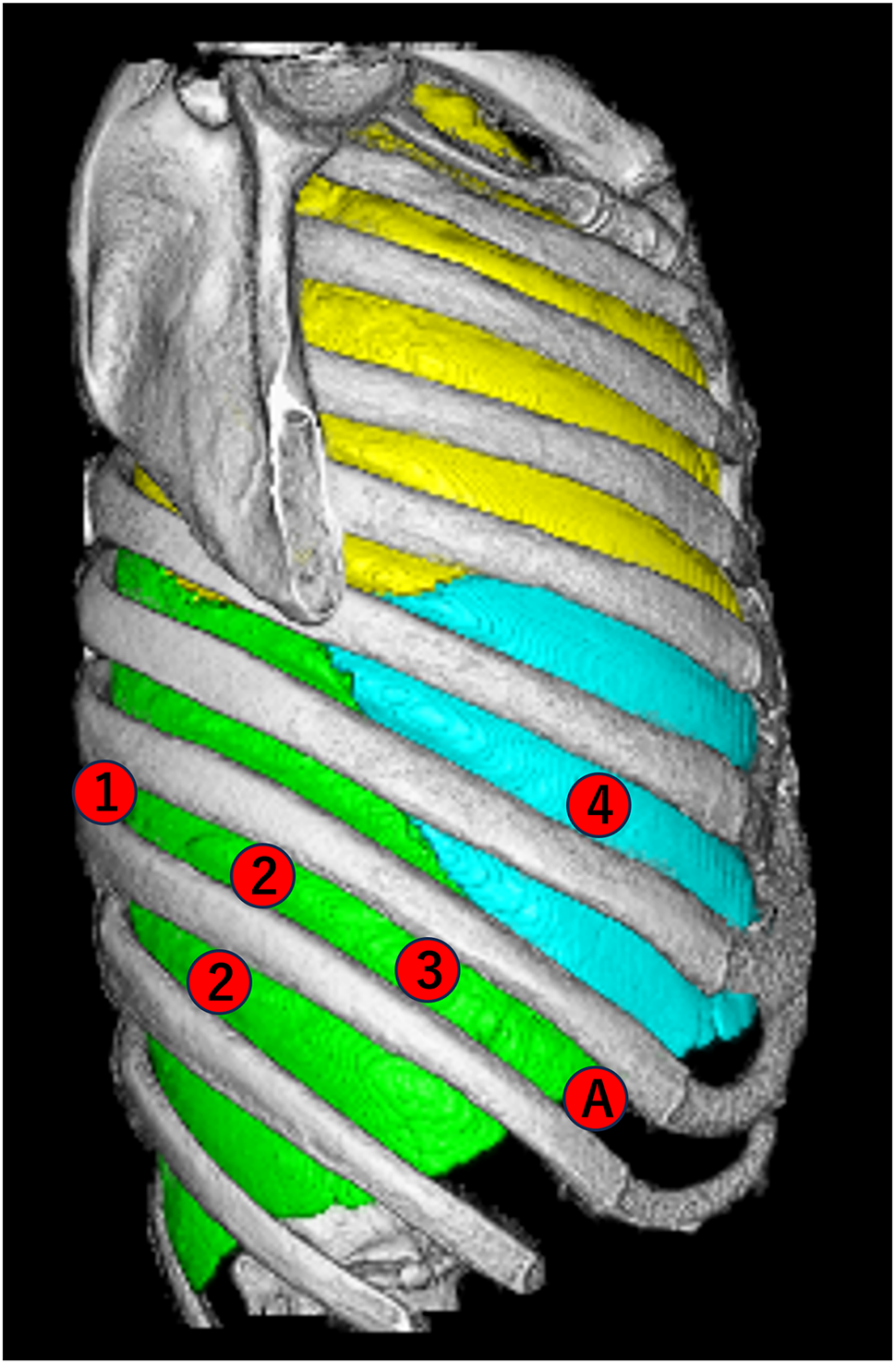
This illustration demonstrates an example of port placement for right-sided surgery. The camera port (③) is positioned in the 8th intercostal space at the midaxillary line. Two da Vinci ports (①, ④) are placed in the 8th intercostal space dorsally and the 6th intercostal space at the anterior axillary line, respectively. Depending on the patient's body size, an additional da Vinci port (②) is inserted in either the 8th or 9th intercostal space at the posterior axillary line. An AirSeal port (A) for CO2 insufflation and assistant access is located in the 8th intercostal space at the anterior axillary line

RATS was performed using the da Vinci Xi Surgical System (Intuitive Surgical, Inc, Sunnyvale, CA) under CO_2_ insufflation with a pressure of 5–8 mmHg. A camera port was placed in the mid-axillary line at the 8th intercostal space, and an assist port was placed in the anterior axillary line at the 8th intercostal space using an AirSeal port (@CONMED). In addition, three ports were typically placed on the 6th and 8th intercostal spaces, resulting in a configuration of four robotic arm ports and one assist port (Fig. 1). In contrast, VATS was performed with three or four small incisions at the discretion of the surgeon. Pulmonary vessels and bronchi were divided using an automatic stapler depending on the surgeon's preference and intraoperative circumstances. Lymph node dissection was performed selectively (ND2a-1) for both RATS and VATS, except in cases with pure ground-glass opacities.

Data on patient demographics, intraoperative parameters, surgical approaches, definitive procedures, perioperative outcomes, and postoperative outcomes were retrospectively collected from electronic medical records and the thoracic surgery department’s database. Patients were divided into the RATS and VATS groups, and their baseline characteristics were compared. The location of the tumor was defined as "peripheral" if the tumor center was located in the outer half of the lung field, with the hilum as the center, and "central" if it was located in the inner half. Surgeon experience was defined as the number of years since the surgeon had obtained their medical license. Propensity score matching was performed to balance the baseline characteristics between the groups. The perioperative, postoperative, and long-term outcomes were compared between the matched cohorts.

Perioperative and postoperative outcomes included operative time, intraoperative blood loss, number of staples used, rates of vascular and bronchial injury, conversion to open thoracotomy, upstaging from cN0 to pN1/2, incidence of postoperative complications (Clavien-Dindo grade ≥ III), postoperative drainage duration, postoperative length of hospital stay, 30-day reoperation rate, and 30-day readmission rate. Long-term outcomes were assessed using the 3-year overall survival (OS) and 3-year disease-free survival (DFS), with the observation starting date set as the date of surgery.

Recurrence was defined as recurrence confirmed by imaging or clinical recurrence based on worsening symptoms without confirmation by imaging. Local recurrence included recurrence of the primary lesion, resection margin (lung parenchyma margin and bronchial margin), ipsilateral hilar lymph nodes, ipsilateral mediastinal lymph nodes, contralateral mediastinal lymph nodes, contralateral hilar lymph nodes, ipsilateral intrapulmonary metastasis, ipsilateral pleural effusion, and ipsilateral pleura. Distant metastases included recurrences in the contralateral lung, contralateral pleural effusion, contralateral pleura, brain, bone, adrenal gland, liver, supraclavicular lymph nodes, pericardium, pericardial effusion, and other sites. Staging was reported based on the 8th edition of the TNM classification for lung cancer. Prolonged postoperative air leak was defined as an air leak persisting for seven days or longer or one that required pleurodesis or surgical intervention.

All statistical analyses were performed using EZR (version 1.67; Saitama Medical Center, Jichi Medical University, Saitama, Japan), a graphical user interface for R (version 4.3.1; The R Foundation for Statistical Computing, Vienna, Austria). Continuous variables were summarized using medians and interquartile ranges (IQRs), and comparisons were made using the Mann–Whitney U test. Categorical variables were summarized using frequencies and percentages, and comparisons were made using the chi-squared test or Fisher's exact test. Furthermore, to adjust for imbalances in patient backgrounds, propensity score matching was used to match patient backgrounds between the RATS and VATS groups as closely as possible. The following factors were used to create the propensity score: sex, age, body mass index (BMI), charlson comorbidity index (CCI), smoking status, %forced expiratory volume (%FEV), %diffusing capacity of the lungs for carbon monoxide (%DLCO), tumor location, lobe, lymph node dissection, maximum tumor diameter, consolidation/tumor (C/T) ratio, and pure solid tumor. The propensity score was created using a logit model and executed using the nearest neighbor method with 1:1 matching performed using a caliper value of 0.2. The balance of covariates after matching was assessed using standardized differences. All p values were two-sided, and statistical significance was defined as p < 0.05.

## Results

### Patient background characteristics

This study included 375 patients, with a median follow-up period of 45.5 months. Of these, 225 patients (60%) underwent RATS, and 150 patients (40%) underwent VATS. Patients who underwent RATS had a significantly higher %DLCO (p = 0.005), lower prevalence of previous cardiac disease (p = 0.025), smaller tumor C/T ratio (p = 0.002), lower proportion of pure solid tumors (p = 0.02), and higher number of years of experience (p < 0.001) than those who underwent VATS (Table 1).

**Table 1:** Patient characteristics

	Group	RATS 225	VATS 150	p value
Gender (%)	Male	112 (49.8)	73 (48.7)	0.916
Age (median [IQR])		71 [64–75]	71 [65–76]	0.779
Body mass index (median [IQR])		22.5 [20.3–25.0]	22.7 [20.6–24.6]	0.874
%FEV1 (median [IQR])		102.2 [89.2–113.0]	96.9 [85.2–109.4]	0.063
%DLCO (median [IQR])		107.7 [96.1–120.7]	101.0 [89.7–114.6]	0.005
Smoking status (%)	Never Smoker	102 (45.3)	63 (42.0)	0.596
CCI (median [IQR])		0 [0–1]	0 [0–2]	0.825
Diabetes mellitus (%)		48 (21.3)	22 (14.7)	0.136
Cardiovascular disease (%)		6 (2.7)	12 (8.0)	0.025
History of malignancy (%)		38 (16.9)	30 (20.0)	0.495
Interstitial pneumonia (%)		11 (4.9)	14 (9.3)	0.093
Tumor laterality (%)	Right	163 (72.4)	108 (72.0)	1.000
Tumor location (%)	Peripheral	177 (78.7)	120 (80.0)	0.796
Lobe (%)	Right upper lobe	95 (42.2)	63 (42.0)	0.692
	Right middle lobe	26 (11.6)	12 (8.0)	
	Right lower lobe	42 (18.7)	33 (22.0)	
	Left upper lobe	38 (16.9)	29 (19.3)	
	Left lower lobe	24 (10.7)	13 (8.7)	
Lymph node dissection (%)	ND1	33 (14.7)	29 (19.3)	0.257
	ND2	192 (85.3)	121 (80.7)	
Maximum tumor size. preoperative mm (median [IQR])		24 [18–31]	21 [17–29]	0.055
Solid component size. preoperative mm (median [IQR])		17 [9–24]	17 [11–24]	0.298
C/T ratio (median [IQR])		0.80 [0.40–1.00]	1.00 [0.60–1.00]	0.002
Pure solid tumor (%)		90 (40.0)	79 (52.7)	0.020
Clinical stage (%)	0	15 (6.7)	4 (2.7)	NA
	IA1	53 (23.6)	28 (18.7)	
	IA2	79 (35.1)	64 (42.7)	
	IA3	45 (20.0)	32 (21.3)	
	IB	21 (9.3)	17 (11.3)	
	IIA	7 (3.1)	2 (1.3)	
	IIB	2 (0.9)	3 (2.0)	
	IIIA	3 (1.3)	0	
Surgeons' years of experience (median [IQR])		20 [16–22]	14 [12–15]	< 0.001

After propensity score matching, 274 patients were selected, of which 137 underwent RATS, and 137 underwent VATS. Before propensity score matching, there were significant differences between the RATS and VATS groups in the %DLCO, history of cardiac disease, tumor C/T ratio, and proportion of pure solid lesions. However, propensity score matching successfully mitigated these differences. Following matching, there were no significant differences in any other baseline patient characteristics between the two groups. An exception was the surgeons' years of experience, with a median of 20 (IQR 16–23) years in the RATS group and 14 (IQR 13–15) years in the VATS group (p < 0.001, SMD 1.265) (Table 2).

**Table 2:** Patient characteristics after propensity matching

	Group	RATS 137	VATS 137	p value	SMD
Gender (%)	Male	71 (51.8)	66 (48.2)	0.629	0.073
Age (median [IQR])		72 [64–77]	71 [64–76]	0.349	0.058
Body mass index (median [IQR])		22.3 [20.2–24.9]	22.6 [20.6–24.8]	0.780	0.021
%FEV1 (median [IQR])		98.5 [88.2–110.7]	97.5 [87.9–110]	0.848	0.037
%DLCO (median [IQR])		104.2 [90.6–117]	103.5 [90.3–115.3]	0.641	0.038
Smoking history (%)	Never Smoker	76 (55.5)	76 (55.5)	1.000	< 0.001
CCI (median [IQR])		0 [0–2]	0 [0–2]	0.521	0.016
Diabetes mellitus (%)		30 (21.9)	21 (15.3)	0.214	0.169
Cardiovascular disease (%)		5 (3.6)	11 (8.0)	0.196	0.188
History of malignancy (%)		29 (21.2)	27 (19.7)	0.881	0.036
Interstitial pneumonia (%)		10 (7.3)	12 (8.8)	0.825	0.054
Tumor laterality (%)	Right	112 (82.4)	101 (67.3)	0.146	0.214
Tumor location (%)	periphelar	106 (77.4)	111 (81.0)	0.552	0.090
Lobe (%)	Right upper lobe	56 (40.9)	60 (43.8)	0.855	0.140
	Right middle lobe	17 (12.4)	12 (8.8)		
	Right lower lobe	26 (19.0)	29 (21.2)		
	Left upper lobe	26 (19.0)	26 (19.0)		
	Left lower lobe	12 ( 8.8)	10 (7.3)		
Lymph node dissection (%)	ND1	24 (17.5)	24 (17.5)	1.000	< 0.001
	ND2	113 (82.5)	113 (82.5)		
Maximum tumor size. preoperative mm (median [IQR])		23 [18–29]	22 [17–29]	0.482	0.051
Solid component size. preoperative mm (median [IQR])		19 [14–26]	17 [11–24]	0.089	0.181
C/T ratio (median [IQR])		1.00 [0.70–1.00]	1.00 [0.60–1.00]	0.261	0.153
Pure solid tumor (%)		75 (54.7)	69 (50.4)	0.545	0.088
Clinical stage (%)	0	2 (1.5)	4 (2.9)	0.709	0.277
	IA1	20 (14.6)	26 (19.0)		
	IA2	59 (43.1)	59 (43.1)		
	IA3	34 (24.8)	28 (20.4)		
	IB	17 (12.4)	15 (10.9)		
	IIA	2 (1.5)	2 (1.5)		
	IIB	1 (0.7)	3 (2.2)		
	IIIA	2 (1.5)	0		
Surgeons' years of experience (median [IQR])		20 [16–23]	14 [13–15]	< 0.001	1.265

### Perioperative outcomes

Table 3 shows the perioperative outcomes. Patients who underwent RATS had a longer operative time (180 [155–213] vs. 144 [123–163], p < 0.001), less blood loss (5 [1–20] vs. 12 [3–40], p = 0.005), and lower rate of conversion to open thoracotomy (1 [0.7%] vs. 10 [7.4%], p = 0.01) than those who underwent VATS. There was no significant difference in the overall incidence of major postoperative complications (Clavien-Dindo grade ≥ III) between the two groups. However, the incidence of prolonged postoperative air leak was significantly higher in the RATS group than in the VATS group (13 [9.5%] vs. 3 [2.2%], p = 0.018), resulting in a significantly longer duration of chest tube placement in the RATS group (2 [2–5] vs. 2 [2, 3], p = 0.018). There were no significant differences between the groups in the postoperative length of stay, 30-day re-operation rate, or 30-day re-admission rate.

**Table 3:** Perioperative outcomes

	RATS 137	VATS 137	p value
Operative time, min (median [IQR])	180 [155–213]	144 [123–163]	< 0.001
Console time, min (median [IQR])	140 [115–171]	NA	NA
Bleeding, ml (median [IQR])	5 [1–20]	12 [3–40]	0.005
Number of staples (median [IQR])	6 [5–8]	6 [4–7]	0.067
Drainage duration, days (median [IQR])	2 [2–5]	2 [2, 3]	0.018
Intraoperative injury (%)	8 (5.8)	12 (8.8)	0.487
Pulmonary artery injury (%)	4 (2.9)	11 (8.0)	0.069
Convert to thoracotomy (%)	1 (0.7)	10 (7.3)	0.010
Postoperative complication ≥ G3 (%)	17 (12.4)	12 (8.8)	0.433
Postoperative prolonged air leak G3	13 (9.5)	3 (2.2)	0.018
Postoperative length of stay, days (median [IQR])	5 [5–7]	5 [5, 6]	0.223
30-day re-operation (%)	2 (1.5)	5 (3.6)	0.447
30-day re-hospitalization (%)	2 (1.5)	5 (3.6)	0.447

**Fig. 2:**
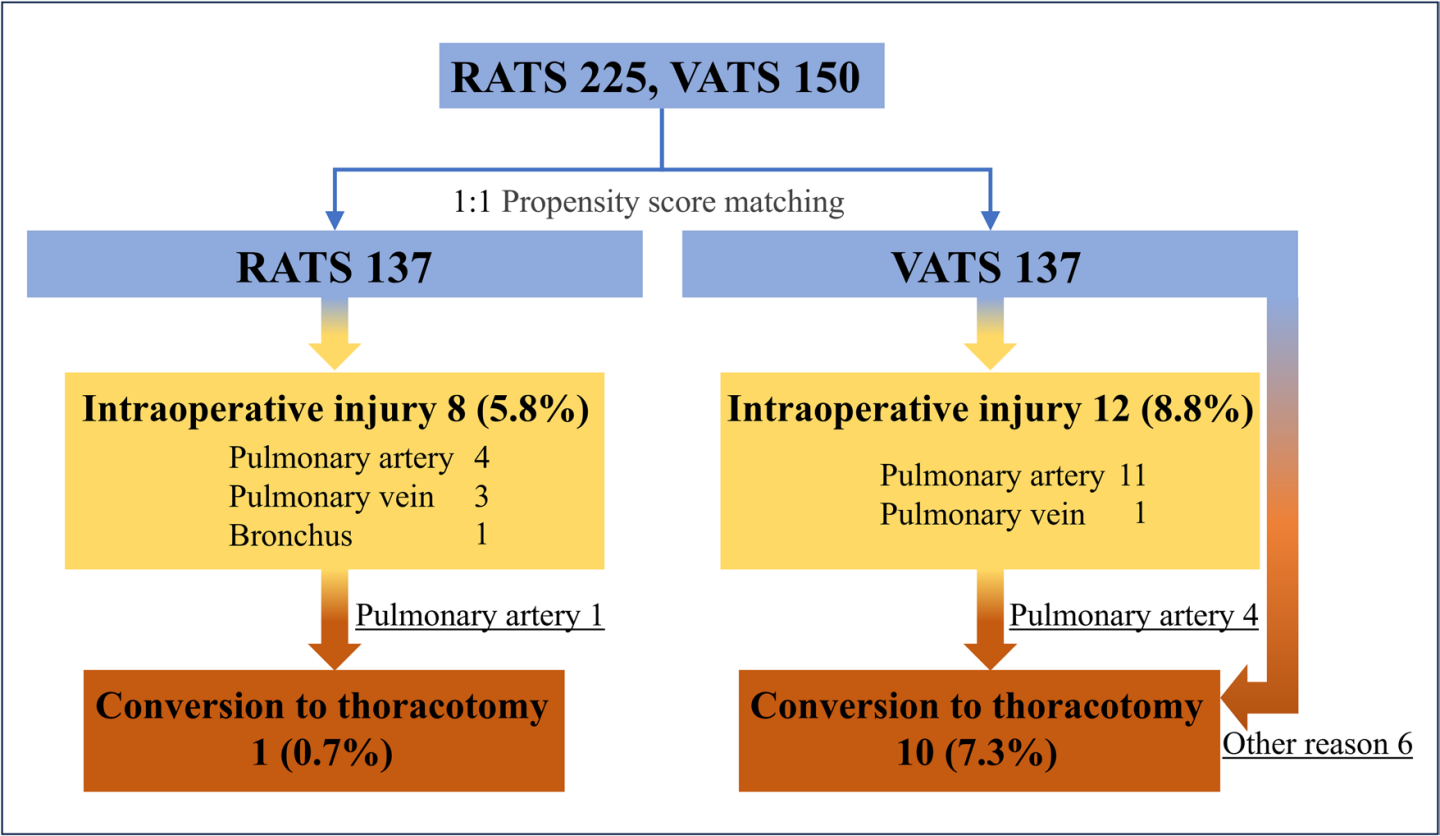
Intraoperative injury cases and flowchart of thoracotomy conversion cases. Underlines indicate the reason for conversion to thoracotomy. One case of RATS conversion to thoracotomy was due to pulmonary artery injury. Of the 10 cases of VATS conversion to thoracotomy, 4 were due to pulmonary artery injury and 6 were due to other reasons

Figure 2 illustrates the flowchart of intraoperative injuries and subsequent conversion to open thoracotomy. Although the overall intraoperative injury rates for vessels and bronchi were comparable between the groups (8 [5.8%] vs. 12 [8.8%], p = 0.487), pulmonary artery injuries were more frequent in the VATS group than in the RATS group (4 [2.9%] vs. 11 [8.0%], p = 0.069). While pulmonary artery injuries accounted for half of the intraoperative injuries in the RATS group, all but one of the intraoperative injuries in the VATS group were due to pulmonary artery injury (Table 4). In the RATS group, the single conversion to thoracotomy was due to pulmonary artery injury (Table 5).

**Table 4:** Intraoperative injury details

	RATS 8	VATS 12
Pulmonary artery	4 (50.0)	11 (91.7)
Pulmonary vein	3 (37.5)	1 (8.3)
Bronchus	1 (12.5)	0

**Table 5:** Reasons for conversion to thoracotomy

	RATS 1	VATS 10
Pulmonary artery injury	1 (100)	4 (40)
Lymph node adhesion to vessels	0	3 (30)
For closure of air leak	0	1 (10)
Incomplete fissure	0	1 (10)
Dense pleural adhesion	0	1 (10)

To evaluate the impact of surgeon experience on perioperative outcomes in the RATS group, patients undergoing RATS were stratified into groups based on the surgeon's prior experience with RATS lung resection, as follows: ≤ 5, > 5, ≤ 10, > 10, ≤ 15, > 15, ≤ 20, and > 20 procedures. Perioperative outcomes were compared between these groups. While a trend towards a longer console time was observed in the group with ≤ 5 prior RATS lung resections, this did not reach statistical significance (p = 0.069). No significant differences in perioperative outcomes were found among the other groups (Supplementary Tables 1, 2, 3, and 4).

### Postoperative outcomes

The postoperative outcomes are shown in Table 6. The proportion of patients with pN2 was significantly higher in the RATS group than in the VATS group, resulting in a greater proportion of patients with pathological stage III disease (11 [8.0%] vs. 7 [5.1%], p = 0.031). However, no significant differences were found in upstaging from clinical cN0 to pN1 or pN2, or in the final pathological diagnosis between the two groups.

**Table 6:** Postoperative outcomes

	Group	RATS 137	VATS 137	p.value
Pathological maximum diameter mm (median [IQR])		20 [16–26]	20 [15–25]	0.489
Pathological invasive diameter mm (median [IQR])		19 [15–25]	18 [13–24]	0.214
Pathological C/T ratio (median [IQR])		1.00 [0.90–1.00]	1.00 [0.83–1.00]	0.157
pN (%)				0.015
	N0	126 (92.0)	130 (94.9)	
	N1	1 (0.7)	5 (3.6)	
	N2	10 (7.3)	2 (1.5)	
Nodal upstaging cN0 → pN1or2 (%)		11 (8.0)	7 (5.1)	0.465
pStage (%)				0.031
	0-IIB	126 (92.0)	134 (97.8)	
	IIIA, IIIB	11 (8.0)	3 (2.2)	
Tumor histology (%)				0.41
	Adenocarcinoma	115 (83.9)	121 (88.3)	
	Squamous	15 (10.9)	15 (10.9)	
	Large cell	2 (1.5)	1 (0.7)	
	Carcinoid	3 (2.2)	0	
	Adenosquamous	1 (0.7)	0	
	LCNEC	1 (0.7)	0	
Recurrence (%)		22 (16.1)	16 (11.7)	0.382
Recurrence site (%)				0.21
	Local	12 (54.5)	6 (37.5)	
	Distant	3 (13.6)	5 (31.2)	
	Distant + Local	7 (31.8)	5 (31.2)	
Death (%)		10 (7.3)	15 (10.9)	0.402
Cause of death (%)				0.073
	Lung cancer	6 (60.0)	6 (40.0)	
	Other cancer	4 (40.0)	3 (20.0)	
	Other	0	6 (40.0)	

### Long-term outcomes

**Fig. 3:**
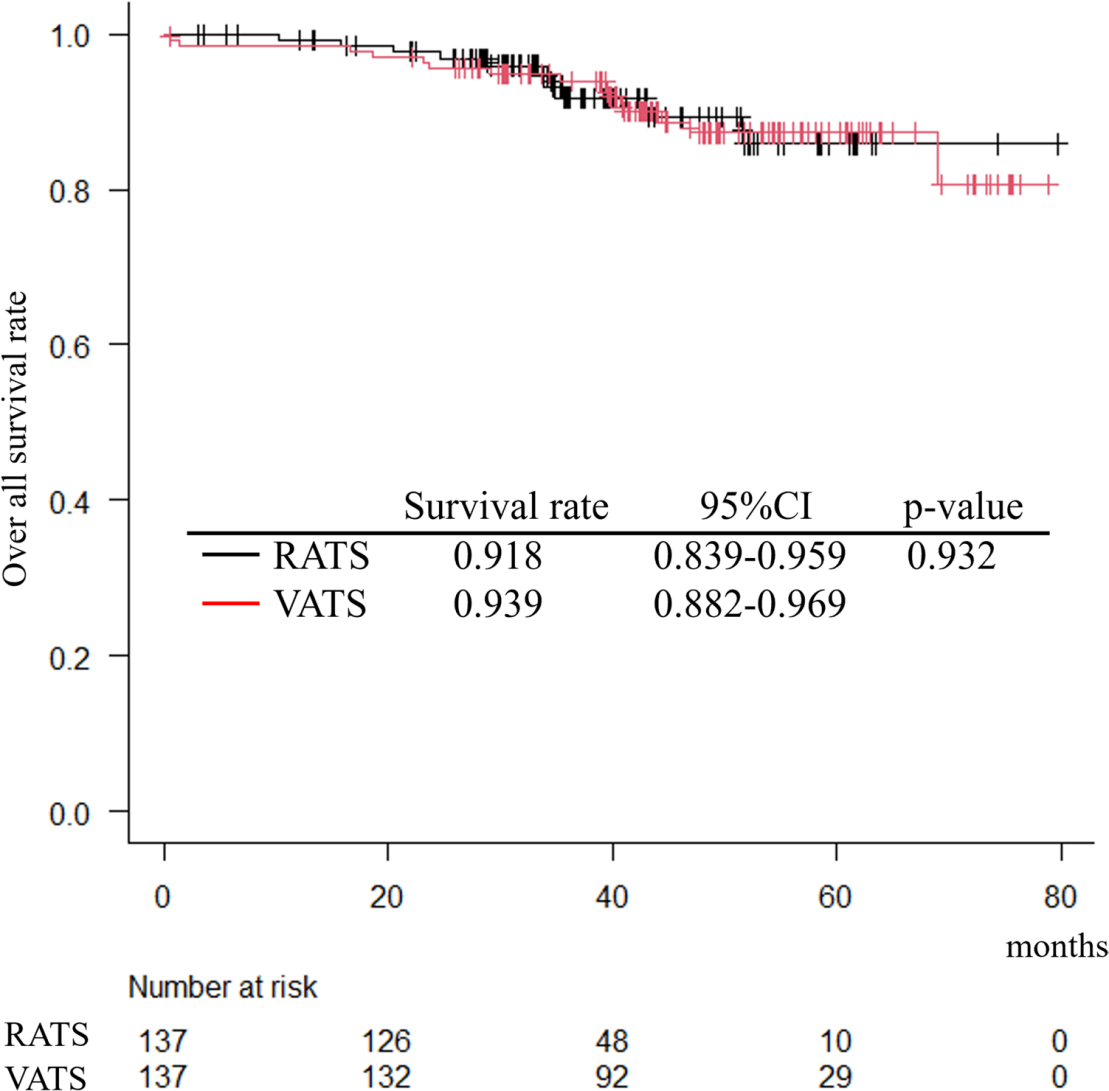
The 3-year survival rate was 0.918 (95% confidence interval 0.839–0.959) for RATS and 0.939 (95% confidence interval 0.882–0.969) for VATS, with no significant difference observed between the two groups (p = 0.932). The median survival time was not reached in either group

The 3-year OS did not differ significantly between the RATS and VATS groups (91.8% vs. 93.9%, p = 0.932) (Fig. 3). There was no marked difference in the distribution of the causes of death.

**Fig. 4:**
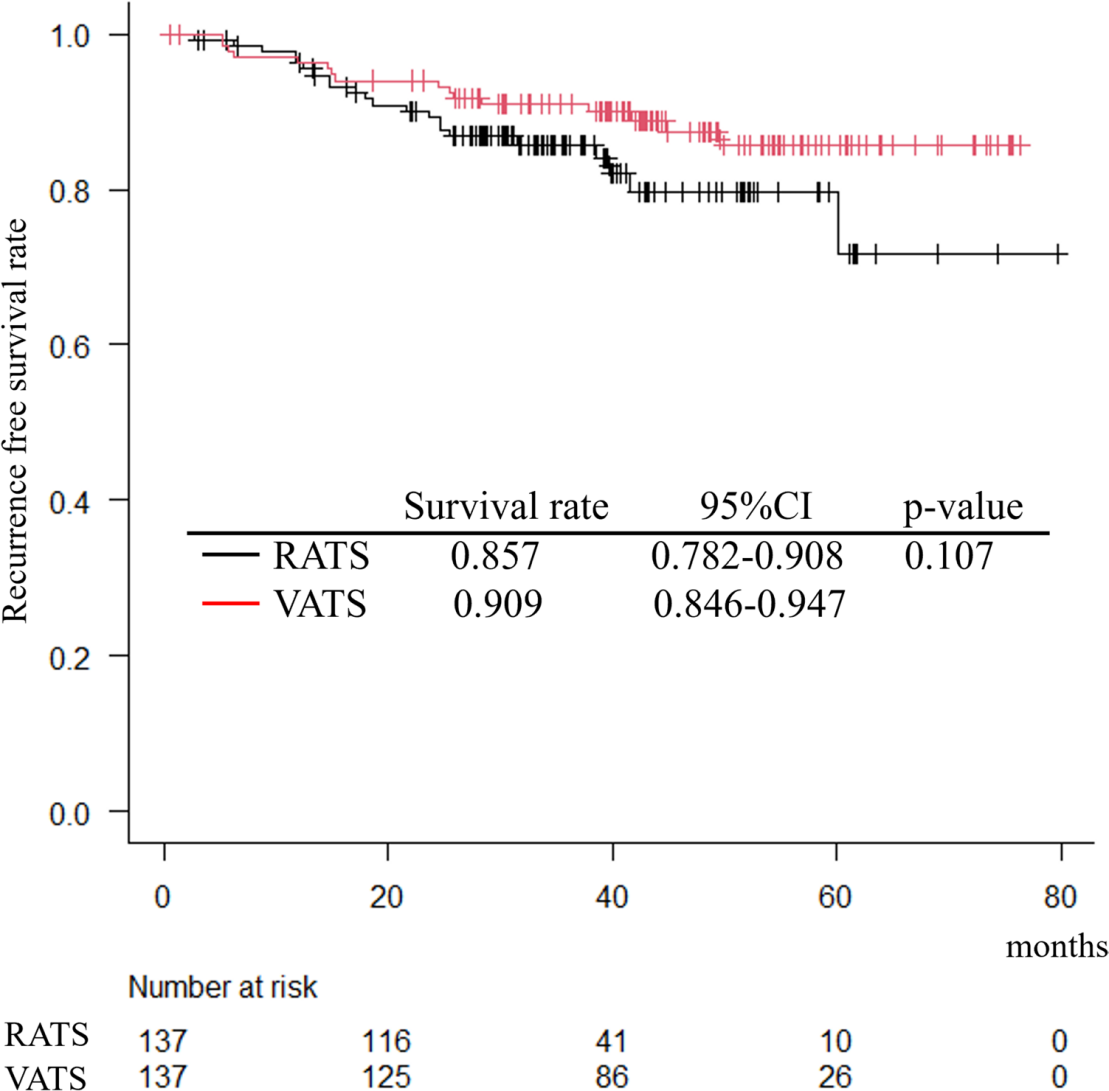
The 3-year disease-free survival rate was 0.857 (95% confidence interval 0.782–0.908) for RATS and 0.909 (95% confidence interval 0.846–0.947) for VATS, with no significant difference observed between the two groups (p = 0.107). The median survival time was not reached in either group

The 3-year DFS did not differ significantly between the RATS and VATS groups (85.7% vs. 90.9%, p = 0.107) (Fig. 4). There was no marked difference in the distribution of the recurrence patterns.

## Discussion

In this study, we compared RATS and VATS for patients with lung cancer who were deemed suitable for lobectomy. The results showed that the long-term outcomes, including three-year survival rates, were comparable between the RATS and VATS groups. This finding is consistent with those of previous meta-analyses and reports from single institutions [2, 4, 6, 7, 9–14]. Similar to previous reports, there was no marked difference in the upstaging rate from cN0 to pN1/2 between the two groups, suggesting that RATS can achieve radicality comparable to VATS [15, 16].

Compared to VATS, RATS has a longer operative time but less blood loss. This suggests that superior maneuverability and high-definition vision of RATS may contribute to more precise surgical manipulation and better bleeding control. However, RATS lacks tactile feedback, necessitating meticulous dissection of the blood vessels and careful handling of the lungs, which may contribute to the longer operative time observed in RATS. In the future, advancements in robotic technology that enable tactile feedback during RATS may prove beneficial in reducing the operative time and further enhancing bleeding control.

The conversion rate from RATS to open thoracotomy varies considerably across reports [1–7]. In our study, RATS had a significantly lower conversion rate to open thoracotomy than VATS. This result is consistent with findings from other studies [1, 4, 5, 7]. There are two possible reasons for this. First, the incidence of pulmonary artery injury was higher in the VATS group than in the RATS group. In our study, the reasons for conversion to open thoracotomy in VATS were emergency thoracotomy due to pulmonary artery injury in 4 out of 10 cases (40%) and maneuverability issues in the others. Conversely, the incidence of pulmonary artery injuries in RATS was low (n = 4), with only 1 case requiring conversion to open thoracotomy due to high urgency. Furthermore, there were no instances in which conversion to open thoracotomy was necessary due to maneuverability issues.

The advantages of high-definition vision and the free maneuverability of forceps with articulated joints in RATS may have resolved various difficult situations and problems that could not be addressed with VATS. Lymph nodes adhering to vessels pose a technical challenge and increase the risk of conversion to emergency or prophylactic open thoracotomy due to vascular injury. RATS, with its enhanced dexterity, precision, and magnified vision, allows for meticulous dissection. For example, even if a lymph node cannot be dissected from a vessel, it is easier with RATS than with VATS to dissect the lung parenchyma, expose the vessel more peripherally, and divide it at a location that avoids the adhered lymph node. Our experience suggests that, at least in our institution, the advantages of magnified vision in RATS outweigh the disadvantages of the lack of tactile feedback when handling vessels, resulting in a reduced conversion rate. Second, the decision to convert to thoracotomy in RATS is significantly influenced by not only the surgeon's experience but also the assistant’s proficiency. At our institution, multiple robotic surgeons during the study period also participated as assistants in other RATS procedures. This means that the assistants were highly skilled and possessed an advanced understanding of the unique movements and blind spots associated with robotic surgery, as they themselves were experienced robotic surgeons. Furthermore, in RATS, in which the surgeon cannot immediately intervene, the assistant's experience is crucial for proceeding with the operation without anxiety about conversion to open thoracotomy. The assistant must be capable of effectively managing emergencies. This may have contributed to the low conversion rate of RATS in our institution.

In our study, while RATS demonstrated a trend toward a lower rate of intraoperative pulmonary artery injury than VATS, the difference was not statistically significant (4 [2.9%] vs. 11 [8.0%], p = 0.069). However, once pulmonary artery injury occurred, the conversion rate to open thoracotomy was similar between the approaches: 25% (1 of 4 cases) for RATS and 36% (4 of 11 cases) for VATS. This suggests that if pulmonary artery hemorrhaging occurs, conversion to open thoracotomy may be necessary with a comparable frequency in both RATS and VATS to ensure safe completion of the operation. This result is consistent with previous studies reporting higher in-hospital mortality in patients undergoing lung surgery who required emergent conversion to open thoracotomy than in those who underwent complete minimally invasive surgery [5]. Therefore, to minimize the need for emergency conversion due to bleeding, it may be necessary in the future to establish clear criteria for conversion, such as a time limit for dissecting lymph nodes adhered to vessels.

As previously reported [17], the incidence of prolonged postoperative air leaks was higher in the RATS group than in the VATS group. Although the reason for the increased incidence of prolonged air leak after RATS could not be elucidated in a previous study, the incidence of prolonged postoperative air leak after VATS at our institution was lower than that in other reports, whereas the incidence of prolonged air leak after RATS was comparable to other published data. A significantly higher incidence of unexpected postoperative air leaks was observed in the RATS group than in the VATS group. This finding suggests that missed air leaks during the intraoperative sealing test may have contributed to this finding. Many of these patients underwent pleurodesis with OK-432 intrapleural administration, which was the main cause of Clavien-Dindo classification grade ≥ III complications in RATS. There were no marked differences in the length of hospital stay, 30-day re-operation rate, or 30-day re-admission rate. Based on our findings and those of previous reports, RATS appears to offer postoperative and long-term outcomes comparable to those of VATS. A future challenge to be addressed is the comparison of the postoperative quality of life between the two groups. Cheng et al. used the QoR-15 scale to show that RATS has better postoperative recovery than VATS [18]. Lan et al. showed that RATS was associated with a lower rate of postoperative dysfunction in the first seven days after surgery than VATS [19]. In our study, we were unable to compare these items because patient pain and recovery data were unavailable.

The median follow-up period was 44.5 months, and the 3-year OS and DFS rates were calculated. There were no significant differences in either the OS or DFS between the two groups, although the RATS group tended to show a lower 3-year DFS than the VATS group. This may be attributed to the significantly higher proportion of pStage III patients in the RATS group. Although further investigation with a larger sample size is necessary, these findings suggest that the surgical approach does not significantly affect the long-term outcomes.

Several limitations associated with the present study warrant mention. First, this was a single-center retrospective cohort study with a limited number of cases. However, the use of propensity score matching to adjust for imbalances in patient backgrounds is believed to yield more reliable results. Second, in both the RATS and VATS groups, the surgeons were not fixed, and differences in skill levels may have been reflected in the results. Because of the Japanese system, in which RATS surgeons are limited to board-certified members of the Japanese Association for Thoracic Surgery, RATS surgeons in this study had significantly more surgical experience than VATS surgeons. Attempts to match years of experience resulted in a drastically reduced sample size and an uneven distribution of cases among specific surgeons, precluding adjustment for this difference between the two groups. Finally, segmentectomy was covered by insurance in 2020 during the study period. Furthermore, because the results of the JCOG0802/WJOG4607L trial [20] were announced around the same time, segmentectomy is now actively performed for lung cancers with a maximum diameter of ≤ 20 mm. Therefore, some of the patients enrolled in this study may now be eligible for segmentectomy, and the patient population may differ somewhat from the current clinical practice. The accumulation of more cases and further investigations are necessary.

In conclusion, at our institution, RATS lobectomy for lung cancer demonstrated long-term outcomes comparable to those of VATS lobectomy. Although RATS was associated with a longer operative time than VATS, its potential benefits included reduced blood loss and a lower conversion rate to open thoracotomy than VATS.

## Supplementary Information

Below is the link to the electronic supplementary material.

Supplementary file1 (XLSX 11 KB)

Supplementary file2 (XLSX 10 KB)

Supplementary file3 (XLSX 10 KB)

Supplementary file4 (XLSX 10 KB)
